# Exposure to Air Ions in Indoor Environments: Experimental Study with Healthy Adults

**DOI:** 10.3390/ijerph121114301

**Published:** 2015-11-10

**Authors:** Peter Wallner, Michael Kundi, Michael Panny, Peter Tappler, Hans-Peter Hutter

**Affiliations:** 1 Institute of Environmental Health, Center for Public Health, , Medical University Vienna, Kinderspitalgasse 15, Vienna 1090, Austria; E-Mails: peter.wallner4@gmail.com (P.W.); michael.kundi@meduniwien.ac.at (M.K.); michael.panny@wgkk.at (M.P.); 2 Austrian Institute for Healthy and Ecological Building, Alserbachstraße 5, Vienna 1090, Austria; E-Mail: p.tappler@innenraumanalytik.at

**Keywords:** air ions, cognitive performance, heart rate variability, lung function, indoor air, wellbeing

## Abstract

Since the beginning of the 20th century there has been a scientific debate about the potential effects of air ions on biological tissues, wellbeing and health. Effects on the cardiovascular and respiratory system as well as on mental health have been described. In recent years, there has been a renewed interest in this topic. In an experimental indoor setting we conducted a double-blind cross-over trial to determine if higher levels of air ions, generated by a special wall paint, affect cognitive performance, wellbeing, lung function, and cardiovascular function. Twenty healthy non-smoking volunteers (10 female, 10 male) participated in the study. Levels of air ions, volatile organic compounds and indoor climate factors were determined by standardized measurement procedures. Air ions affected the autonomous nervous system (in terms of an increase of sympathetic activity accompanied by a small decrease of vagal efferent activity): In the test room with higher levels of air ions (2194/cm^3^ *vs.* 1038/cm^3^) a significantly higher low to high frequency ratio of the electrocardiography (ECG) beat-to-beat interval spectrogram was found. Furthermore, six of nine subtests of a cognitive performance test were solved better, three of them statistically significant (verbal factor, reasoning, and perceptual speed), in the room with higher ion concentration. There was no influence of air ions on lung function and on wellbeing. Our results indicate slightly activating and cognitive performance enhancing effects of a short-term exposure to higher indoor air ion concentrations.

## 1. Introduction

Air ions are charged particles that are generated by cosmic radiation and radioactive decay in air and ground [1,2]. They are also generated by waterfalls (Lenard effect), friction forces in storms and by lightning [1,3,4]. Measurements of air ions in ambient air showed that concentrations of air ions are varying significantly between different environments. A high concentration of air ions can be found for example close to waterfalls with concentrations up to 10^5^ ions/cm^3^ whereas in urban areas and indoor environments the amounts drop to several hundred ions/cm^3^ or less [5,6,7].

The first observations of the existence of air ions in natural environments were reported at the end of the 19th century [3]. For many decades there has been a debate about potential biological effects of air ions and of indoor air ionizers. Some studies showed that higher concentrations of (negative) air ions may have, inter alia, a positive influence on alertness and cognitive performance [8,9,10]. With regard to mood, no consistent influence of positive or negative air ions has been observed in other studies [5]. However, there are indications that artificially produced higher concentrations of negative air ions may be effective in the treatment of depressions and seasonal affective disorder [11,12]. A recent meta-analysis concluded that negative air ionization was associated with lower depression ratings [5]. High densities of negative air ions resulted in a change in serotonin levels in the brains of rats and mice [13,14,15]. Bailey and Charry [16], however, found no effect of exposure to air ions on the concentration or turnover of serotonin in rats.

Whereas ionized waterfall aerosol had a beneficial effect on asthma symptoms, lung function, and airway inflammation [6], a systematic Cochrane review stated [17] that room air ionizers did not improve pulmonary function of patients with asthma. In a review of 23 studies (published from 1933 to 1993) Alexander *et al.* [18] found “no persuasive evidence” for effects of air ions on respiratory function. They summarize, however, that some studies reported beneficial effects of negative air ions, while other studies found mild adverse effects of positive ions [18]. Using ergometry in healthy male subjects, sudden introduction or removal of negative air ions induced increases in ventilation rate, breathing equivalent, and cardiac frequency [19]. Herrington [20] and Albrechtsen *et al.* [21] found no effect on heart rate, whereas Yaglou *et al.* [22] suggested that their data indicate a normalizing effect of air ions on pulse rate (and other physiological functions). Generally, it remains unclear, whether effects are due to increased concentrations of negative air ions or to increased total ion levels.

In this study, effects of increased indoor levels of air ions on wellbeing, cognitive performance, lung function, and cardiovascular parameters (heart rate variability) were investigated. The increase in air ion concentrations was not achieved by an ionizer, but by a mineral based wall paint. The wall paint under study was a development intended to counteract the depletion of ions indoors. While earlier approaches to increase indoor ion concentration (e.g., by corona discharge) had the disadvantage to create ozone the wall paint has no such shortcomings. The product was investigated concerning its potential to generate air ions by the German Fraunhofer Institute of Building Physics [23]. 

## 2. Experimental Section 

### 2.1. Participants and Procedure

A randomized double-blind crossover experiment on 20 healthy non-smoking volunteers (10 female, 10 male; age groups 20–35y—5 male, 5 female and 40–55y—5 male, 5 female) was carried out in summer 2010. Participants were recruited through snowball sampling.

In two experimental sessions of this field investigation each volunteer underwent the same standardized program in each of two rooms. The experiment (with one volunteer per session) had a duration of two hours, each of the two sessions on the same day of the week and at the same time of the day, one week apart. Half of the volunteers started the program in room A, the other in room B. Room B had higher air ion concentrations (see 2.2.). Participants were instructed to refrain from coffee drinking and not to eat two hours before the experiment and to avoid heavy physical exercise.

Upon their arrival, the participants were instructed about the procedure. After spirometry they were equipped with electrocardiography (ECG) electrodes and recorders. During the two hour session participants started with a period of standardized activity (crossword puzzle) for 25 min. Then they completed, in order, the Self Condition Scale by Nitsch (5 min) [24] followed by the general cognitive performance test by Horn (subtests 1–4) (10 min) [25], again followed by the Self Condition Scale (5 min). After that the second period of standardized activity (jigsaw puzzle) was initiated and lasted 55 min. In the next step the second part of the cognitive performance test by Horn (subtests 7–9, 13, 14) (15 min) and the Self Condition Scale by Nitsch (5 min) was filled in. After leaving the test room spirometry tests were performed again.

The reason for this order was that the participants should first adapt to the experimental situation and relax by doing a crossword. We wanted to ensure to keep the participants busy with some simple activities (puzzle) during the 2 h exposure in order to avoid boredom or stressful thoughts, *etc.,* and to maintain a relaxed and restorative living room situation. The puzzle was introduced to standardize the activity and to keep the volunteers busy with a not too demanding task. The puzzles were 200-piece pictures of animals.

A trained and well experienced study assistant was always present before and during the whole experimental session. She directed the participants from outside of the room via intercom system (plus camera), in order to minimize disturbance (noise, *etc.*) and for adherence to the schedule. 

The volunteers were told that the study is about indoor air quality. All subjects gave their written consent for inclusion before they participated in the study after being informed about the procedure and potential risks in accordance with the standards of the Ethics Committee of the Medical University of Vienna. We informed the subjects in writing that the investigations are non-invasive and pose no health risks. The study was conducted in accordance with the Declaration of Helsinki and the protocol was approved by the Ethics Committee of Vienna (377/2010). 

### 2.2. Experimental Setting

The experiment was conducted in two identical rooms (A and B) in a flat of a tenant house in Vienna, Austria. The building was not on a busy road with a L_den_ in the category 55 to 60 dB according to the noise information system of the city of Vienna and the windows were to the back so that air pollution and noise were attenuated (in addition sound insulating windows were mounted). The rooms were furnished like typical living rooms; one room was painted with a newly developed wall painting to reach a higher concentration of (negative) air ions (room B). There was no recognizable difference between the rooms which could unblind the room with the special mineral interior wall and ceiling paint with silicate binder (Ionit©, Baumit GmbH, Wopfing, Austria). The setting was similar to other studies investigating effects of components of indoor air (e.g., [26]); however, the test rooms in our chamber were not laboratory rooms but identically furnished rooms in an ordinary tenant building.

The mechanisms which lead to higher air ion concentrations have been investigated via environmental scanning electron microscopy, atomic force microscopy, electrostatic force microscopy, and Klevin force microscopy (see Supplementary Material). 

### 2.3. Measurements of Indoor Air Parameters

Air ions, temperature, and humidity were monitored continuously approximately one meter above floor level one hour before and during the tests. Air ions were measured with Ionometer IM806 (Umweltanalytik Holbach GmbH, Wadern, Germany), which counts the number of negative and positive air ions. The measurement interval was 5 s. Results were presented as arithmetic means over the testing period. Furthermore, carbon dioxide concentrations were determined by infrared absorption with testo sensor 535 (testo GmbH, Vienna, Austria).

Volatile organic compounds (VOC) air samples were taken by using adsorption tubes containing a special activated charcoal (Anasorb 747, SKC Limited, Eighty Four, PA, USA). Sample flow rates were about two liters per minute. VOCs were extracted from activated carbon with 1 mL of CS_2_ and analyzed by gas chromatography/mass spectrometry (Shimadzu QP 5000, Scientific Instrument Services,Kyoto, Japan), using a 60 meter fused silica capillary column (HP-VOC) following the Austrian Standard ÖNORM M 5700-2 [27]. As internal standards cyclooctane and toluene-d8 were used.

### 2.4. Cognitive Performance

Cognitive performance was tested by the general cognitive performance test by Horn [25], which is based on Thurstone’s intelligence model. This model describes seven primary mental abilities as basis of the human intelligence. From the test system nine subtests—verbal factor (1,2), reasoning (3,4), space and closure (7–9) and perceptual speed (13,14)—with up to 40 items each were used. Subtest 1 and 2 are assessing verbal fluency and consist of 40 nouns each, with each noun containing a wrong letter which has to be detected. In subtest 3 and 4, (ir)regularities in geometric figures, letters and numbers have to be identified and a series must be completed in each of 40 items. Subtests 7, 8, and 9, each consisting of 40 items, are focusing on spatial perception and closure. Subtest 7 tests spatial rotation, subtest 8 and 9 consist of comparisons between symbols. Raw scores were transformed to C scores (standardization to a mean of 5 and a standard deviation of 2).

### 2.5. Wellbeing

Wellbeing was assessed by the self-condition scale by Nitsch [24]. With this standardized questionnaire the subjects characterize their actual state by 19 attributes (6-step-scale, “does not apply at all—apples fully”) which map motivation and strain. The items belong to six dimensions: readiness for action, readiness for exertion, alertness, state of mood, tension/relaxation and recuperation. 

### 2.6. Lung Function

Lung function of the participants was assessed by spirometry (Masterscope, Viasys Healthcare Inc. San Diego, CA, USA) performed by a trained technician. The calibration of the spirometer was carried out each morning prior to the experiments. Lung function tests were performed according to the protocol of the American Thoracic Society [28]. Both volume (FVC, Forced Vital Capacity, FEV1, Forced Expiratory Volume in the 1st second) and flow parameters (PEF, Peak Expiratory Flow, MEF25, 50, 75, Maximum Expiratory Flow at 25%, 50% and 75% of the FVC and MMEF, Mean Maximum Expiratory Flow) were measured and documented.

### 2.7. Heart Rate Variability (HRV)

With a portable ECG device heart rate and its variability was analysed. Volunteers were equipped with Medilog© AR12 Holter ECG recorders (Schiller, Bar, Switzerland) weighing 75 g. The signal was digitized with a sample rate of 4096 Hz. Recorded data were digitally stored on SD memory cards and afterwards uploaded to a computer for analysis.

The continuous ECG recordings were visually inspected for artefacts and were analysed with the program Medilog Darwin (Schiller, Linz, Austria). Data of all test persons were assembled into one Excel^®^ file and were then imported to Statistica 10.0 (StatSoft (Europe) GmbH, Hamburg, Germany) for further analysis. Spectral analysis was performed in order to analyse the frequency components of the beat-to-beat intervals by using fast Fourier transform with a window width of 128 points. Non-normal beats like extrasystoles were excluded from the analyses. A 20 min period that was as artifact free as possible within the time window 60 to 90 min after start was chosen for the measurement of HRV. 

The HF (high frequency: range 0.15–0.4 Hz) component of HRV represents mainly parasympathetic activation of the autonomous nervous system, the LF (low frequency: range 0.04–0.15 Hz) component is assumed to be influenced mainly by sympathetic activity (Task force of the European Society of Cardiology and the North American Society of Pacing and Electrophysiology) [29].

### 2.8. Statistical Analyses

Data were evaluated by analysis of variance with two factors (sequence: A after B *vs.* B after A and room type: A *vs.* B). Gender and age were included as covariates. Normality of residuals was tested by Kolmogorov-Smirnov tests with Lilliefors’ *p*-values. Homogeneity of variances was tested by Bartlett tests. Comparison of climate and air pollution parameters in room A and B was evaluated using Mann-Whitney U tests. For all tests, p-values below 0.05 were considered significant.

## 3. Results and Discussion

Twenty healthy volunteers, 10 men and 10 women, were included in the study. Average age was 36.5 years. Average concentrations (Table 1) of Volatile Organic Compounds (VOC), as indicator for indoor air quality, were well below the guideline level for indoor environment (1 mg/m^3^) [30] and did not differ significantly between the two test rooms. Average temperatures ranged between 25 °C and 26 °C, humidity between 46% and 48%, showing no significant difference between the two rooms as was the case for CO_2_ levels that were between 400 and 800 ppm, well below the guideline value of 1000 ppm [30]. 

Indoor air ion concentrations were significantly higher in room B than in room A: Total air ion concentration in room B was 2194 per cm^3^ *versus* 1038 per cm^3^ in room A; negative air ion concentrations were 866 per cm^3^ (room B) *vs.* 367 per cm^3^ (room A); positive air ion concentrations were 1328 per cm^3^ (room B) *vs.* 671 per cm^3^ (room A). An overview of the indoor air climate factors and pollutants are given in Table 1.

**Table 1:** Indoor air climate factors (temperature, relative humidity), CO_2_, volatile organic compounds (VOC), formaldehyde and air ions in rooms A and B (arithmetic means).

Factor	Unit	Room A	Room B	*p*-Value
Temperature	°C	25.8	25.3	0.724
Relative humidity	%	48.0	46.3	0.085
CO_2_	ppm	648	624	0.244
VOC total	µg/m^3^	312	357	0.554
Formaldehyde	ppm	0.05	0.05	0.937
Air ions total	ions/cm^3^	1038	2194	<0.001
negative	ions/cm^3^	367	866	<0.001
positive	ions/cm^3^	671	1328	<0.001

CO_2_: Carbon dioxide; VOC: Volatile organic compounds.

In summary, in room B with the higher air ion concentrations, subjects performed better in the cognitive test (Horn’s test). There was no influence of air ions on wellbeing (Nitsch self condition scale) and lung function. With regard to heart rate variability, in room B an increase of low frequency and a decrease of high frequency components were observed, resulting in a significantly higher LF/HF ratio. 

In six of nine subtests of the Horn test participants showed better performances in the room with higher ion concentrations (room B) (Table 2). There were significant differences in the results of three subtests (verbal factor, reasoning, and perceptual speed).

Results of spirometry are shown in Table 3. None of the measured lung function parameters showed a significant difference between rooms. Also, differences between tests before and after the stay in the test rooms were small and not significant.

**Table 2:** Cognitive performance tested by Horn’s test. Means (SD) for rooms A and B (the room with higher air ion concentration). Values are standardized C scores. Higher values define a better cognitive performance. *p*-values from factor room type of analysis of variance.

Factor	Subtest	Room A	Room B	*p*-Value
Verbal Factor	1	3.46 (0.51)	3.24 (0.45)	0.130
	2	3.53 (0.41)	3.86 (0.37)	<0.001
Reasoning	3	6.92 (1.95)	7.51 (1.67)	0.008
	4	7.34 (1.84)	7.16 (1.55)	0.264
Space & Closure	7	6.70 (2.29)	6.84 (2.32)	0.428
	8	5.63 (0.66)	5.87 (0.90)	0.102
	9	7.15 (1.09)	7.07 (1.41)	0.816
Perceptual Speed	13	5.59 (2.17)	6.73 (2.39)	0.042
	14	3.80 (2.18)	4.10 (1.89)	0.481

**Table 3:** Results of ANOVA of spirometric findings.

Parameter	Unit	Room (A *vs.* B)	Before *vs.* After	Difference in Trend (A *vs.* B)
		*p*-value	*p*-value	*p*-value
FVC	mL	0.698	0.600	0.919
FEV1	mL	0.109	0.126	0.272
PEF	mL/s	0.731	0.289	0.675
MEF75	mL/s	0.075	0.274	0.778
MEF50	mL/s	0.244	0.988	0.357
MEF25	mL/s	0.536	0.307	0.464
MMEF	mL/s	0.511	0.448	0.519

FVC: Forced Vital Capacity; FEV1: Forced Expiratory Volume in the 1st second; PEF: Peak Expiratory Flow; MEF25, 50, 75: Maximum expiratory flow at 25, 50 and 75% of FVC; MMEF: Mean maximum expiratory flow.

**Figure 1:**
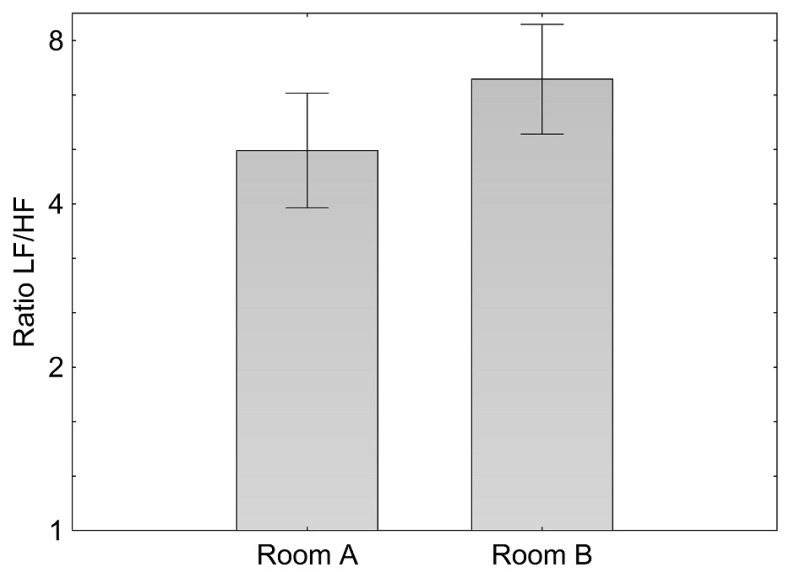
Means and confidence interval of log ratio of the low to the high frequency component (LF/HF) during 20 min standardized activity while seated in room A and B.

With regard to heart rate variability (HRV), a significant difference (*p* = 0.007) in the ratio of low frequency (LF) to high frequency (HF) components was found between the two rooms. In room B (higher air ion concentrations) an increase of the low-frequency (LF) and a decrease of high-frequency (HF) components were observed, resulting in a significantly higher LF/HF ratio (Figure 1).

Effects of exposure to air ions are still a matter of debate. In recent years, there has been a renewed interest in this topic [2,5,6,18]. The aim of our randomized, double-blind, crossover study was to evaluate acute effects of increased concentrations of air ions indoors on wellbeing, cardiovascular function, lung function and cognitive performance. Air ionization (in one of two rooms) was not produced by an ioniser (which may have adverse side effects from increasing ozone levels) but by means of a newly developed wall paint. The resulting air ion concentrations were lower than the concentrations measured close to ionisers or waterfalls but still much higher than typically found in urban indoor environments.

To our knowledge, this was the first study evaluating short term effects of indoor air ion concentrations on HRV in humans. With regard to the LF/HF ratio, which is often suggested to represent sympatho-vagal balance, we found a higher ratio in the room with higher air ion concentration. The effect on HRV may indicate that air ions affected the sympatho-vagal balance which may be due to either a slightly higher sympathetic activity or a slightly diminished vagal activity or both.

This effect on the cardiovascular system could be responsible for the better cognitive performance (in most subtests and with statistical significance in three subtests), as Murray and Russoniello [31] demonstrated that a moderate level of arousal based on increased sympathetic nervous system activity was associated with an improved cognitive performance. But it is also possible that these observed effects are due to independent pathways and consequences of more basic interactions of air ions with body tissues.

In a study on rats, negative air ions were also found to modulate the regulation of autonomic nervous system activity and to influence HRV [32].

In accordance with the conclusions from a recent review we found no effects of air ions on lung function. We also found no influence on wellbeing of test persons which might be related to the short duration of two hours only.

Taking into account that the experiment was conducted under a “real life situation” (identical living rooms as test rooms) while controlling for the most important indoor air quality factors, we think that this setting is a strength of the study and together with the double-blind condition might serve as a new approach in the evaluation of indoor air ions. A limitation is the conduction during one season (summer) only, because physiological as well as psychological function may vary over the year. Also we could not differentiate between effects of positive and negative air ions as the wall paint increases both species. The study was powered to detect rather strong effects only. Therefore, more subtle effects would afford larger sample sizes.

## 4. Conclusions

The present study investigated short-term effects of air ions on physiological and psychological parameters. The results of the presented study do not provide information about any further development of physiological and psychological regulatory mechanisms while being chronically exposed to higher air ions levels.

In our experiments, the elevated air ion levels were produced by a special wall paint. As this is a new method to generate air ions, further studies on the topic with this method are warranted.

## Supplementary Files

Supplementary File 1
