# Supplementary material for: Exposure to Air Ions in Indoor Environments: Experimental Study with Healthy Adults
Source: Int J Environ Res Public Health. 2015 Nov 10;12(11):14301–11. doi: 10.3390/ijerph121114301 (PMC4661648; doi:10.3390/ijerph121114301)
Supplement: Supplementary File 1 [file ijerph-12-14301-s001.pdf]

Supplementary information

Exposure to Air Ions in Indoor Environments: Experimental Study with Healthy Adults

---

"IONIT® Wandcreme" is a silicate and acrylic based interior wall and ceiling paint formulated to produce air ions. This is accomplished by a special combination of minerals such as muscovite, calcite, kaolin and ground semiprecious stones. The size, shape and chemical composition of the mineral particles have an influence on the air-ions generating effect as shown by the Fraunhofer Institute for Building Physics [1]. The product is patented under EP 2 665 780 B1.

The basic effect of passive ionization has been found to originate from (1) morphological characteristics of the surface structure and (2) material properties of the mineral ingredients. The latter is based on the insulating character with high dielectric constants as often found for mineral materials [2]. This favours the physisorption of polar water molecules on the surface by induced dipole formation in the substrate [3]. After this first step, the high number of sharp edges and spikes of the mineralic material is of importance. As the induced dipole momentum increases the electrical field on the edges and spikes more water molecules are attracted and this leads to field induced ionization of the water molecules which then desorb as ionic species from the surface [4]. After such clearance events the induced dipoles in the substrate are neutralized and the process starts again by new water physisorbtion. This process is not exhaustible due to this circular mechanism of action. The electrostatic forces, on the other hand, are much too low to produce ozone or radical oxygen species.

In three experiments applying different settings and conducted at different institutions it has been demonstrated that Ionit® indeed increases the air ion concentration significantly (Table S1). These experiments were conducted at the Fraunhofer Institute for Building Physics (FI), at the Institute of Environmental Medicine and Hospital Hygiene at the Medical Faculty of the University Freiburg (UF) and at our Institute (MUW).

Table S1. (Mean ± Standard Deviation SD) concentration of air ions/cm^3 in different experiments at different institutions in rooms or test chambers applying Ionit® mineral paint or conventional mineral paint.

Institution	Setting	Conventional Mineral Paint		Ionit® Mineral Paint	
		Positive ions	Negative ions	Positive ions	Negative ions
FI	Test chamber	1608 ± 353	1361 ± 328	3096 ± 489	2445 ± 524
UF	Biological exposure chamber	164 ± 92	287 ± 109	4392 ± 361	3750 ± 74
MUW	Living rooms in a flat	671 ± 74	367 ± 32	1328 ± 91	866 ± 77

References

1 Untersuchungen zum Einfluss von Wandbeschichtungen auf die Ionenzahl und das Verhalten von Partikeln in der Raumluft. Available online: http://www.innenraumanalytik.at/pdfs/fraunhofer_ionen.pdf (assessed on 25 August 2015)

2 Kong, L.B.; Li, S.; Zhang, T.S.; Zhai, J.W.; Boey, F.Y.C.; Ma, J. Electrically tunable dielectric materials and strategies to improve their performances. *Prog. Mate.r Sci.* **2010**, *55*, 840–893.
3 Bormashenko, E.Y. *Wetting of Real Surfaces*; Walter de Gruyter: Berlin, Gemany, 2013.
4 Forbes, R.G. Low-macroscopic-field electron emission from carbon films and other electrically nanostructured heterogeneous materials: hypotheses about emission mechanism. *Solid State Electron*. **2001**, *45*, 779–808.

© 2015 by the authors; licensee MDPI, Basel, Switzerland. This article is an open access article distributed under the terms and conditions of the Creative Commons Attribution license (http://creativecommons.org/licenses/by/4.0/).
